# Administration of troxerutin improves testicular function and structure in type-1 diabetic adult rats by reduction of apoptosis

**Published:** 2019

**Authors:** Afsaneh Qadiri, Fariba Mirzaei Bavil, GholamReza Hamidian, Zohreh Zavvari Oskuye, Mahdi Ahmadi, Hajar Oghbaei, Keivan Mehri, Amir Mansour Vatankhah, Rana Keyhanmanesh

**Affiliations:** 1 *Department of physiology, Faculty of medicine, Tabriz University of Medical Sciences, Tabriz, Iran.*; 2 *Student research committee, Tabriz University of Medical Sciences, Tabriz, Iran.*; 3 *Drug Applied Research Center, Tabriz University of Medical Sciences, Tabriz, Iran.*; 4 *Department of Basic Sciences, Faculty of Veterinary Medicine, University of Tabriz, Tabriz, Iran.*; 5 *Tuberculosis and Lung Diseases Research Center, Tabriz University of Medical Sciences, Tabriz, Iran.*

**Keywords:** Diabetes, Troxerutin, Testis, Apoposis, Stress oxidative, Rat

## Abstract

**Objective:**

The glucose-reducing effects of troxerutin was previously proven. This study was conducted to evaluate troxerutin effect on testicular structure and spermatozoid parameters in type-1 diabetic adult male rats.

**Materials and Methods:**

Fifty male Wistar rats were randomly classified into 5 groups as follows: control (C), troxerutin (T), diabetic (DM), troxerutin-treated DM (DT) and insulin-treated DM (DI). Testicular structure, apoptosis, lipid peroxidation and antioxidant activity, and spermatozoid parameters were assessed 4 weeks after initiation of the interventions.

**Results:**

The results revealed that diabetes caused testicular stereological changes and significantly increased blood glucose level, testicular MDA content and apoptosis but decreased insulin level, testicular GPX activity, and sperm parameters compared to controls (p<0.001 to p<0.05). Administration of troxerutin and insulin could significantly reduce blood glucose level and improve testicular MDA content, testicular stereological findings and apoptosis compared to DM group (p<0.001 to p<0.05).

**Conclusion:**

Taken together, troxerutin, comparable to insulin, effectively improved DM-induced testicular dysfunction and sperm parameters in diabetic rats and these effects might be mediated through troxerutin’s anti-apoptotic effects.

## Introduction

The increasing incidence of diabetes mellitus (DM) and its complications has made this disease one of the major health threats to the humans. Chronic increased levels of blood glucose affect all organs, including gonads (Jangir et al., 2014 ▶). Approximately 90% of diabetic patients have abnormalities in sexual functions, including decreased libido as well as impotency and infertility (Ballester et al.,2004 ▶).

Evidence suggests that DM causes excessive production of reactive oxygen species (ROS) and impairment of antioxidant defense system (Amaral et al., 2006 ▶). It was found that human spermatozoa are highly vulnerable to oxidative stress and ROS overproduction in gonads which may play a role in infertility through destruction of sperm membranes and affecting its motility (Karimi et al., 2011 ▶).

Oxidative stress-dependent and -independent apoptosis were found to be involved in DM-induced testicular dysfunction (Kanter et al., 2012 ▶). Similarly, it was shown that injection of streptozotocin and induction of diabetes, led to an increase in germ cell apoptosis and abnormal spermatogenesis (Keyhanmanesh et al., 2018 ▶).

Insulin was shown to facilitate several functions in Sertoli cells, such as free nucleic acids removal, transferrin secretion, DNA and protein synthesis, glycine metabolism, and lactate production (Oliveira et al., 2012 ▶). Also, insulin participates in the differentiation of spermatogonia into primary spermatocytes via insulin-like growth factor (IGF)-1 receptor (Nakayama et al., 2004 ▶).

Testicular tissue studies done in rat models of DM show structural changes, such as decreases in the testicular weight and mass, thickening of the encapsulating structures, decreases in the diameter and height of the germinal epithelium in the seminiferous tubules, increases in the fat droplets and vacuoles in the Leydig cells and the volume of interstitial matrix, and decreases in the number of Leydig cells (Kianifard et al., 2012 ▶).

Several medications have been used to treat DM and reduce its resulting complications. In addition to their positive effects, these medications may render side effects on the reproductive organs. It was found that metformin and glibenclamide, two most commonly used antidiabetic medications, increase lipid peroxidation and impair the antioxidant defense system in the testes, resulting in testicular tissue damage and a significant decrease in the number and motility of the sperms (Adaramoye et al., 2012 ▶).

Troxerutin, also called vitamin P_4_, is a derivative of bioflavonoids, that possesses various biological effects, such as antioxidant, anti-inflammatory, and anti-diabetic characteristics activities (Lu et al., 2011 ▶). The antioxidant features of troxerutin may be due to its impacts on the production of ROS and the activity of antioxidants enzymes (Vinothkumar et al., 2014 ▶). Further, troxerutin increases the overall sensitivity of the cells to insulin (Geetha et al., 2014 ▶). 

Previous studies proved the glucose-reducing effects of troxerutin, but to the best of our knowledge, the effect of this medication on spermatogenesis and testicular function has not been investigated. This study was conducted to evaluate the effect of troxerutin on the structure of testis, spermatozoid parameters, oxidative stress, and apoptosis in the testis of type-1 diabetic adult male rats.

## Materials and Methods

Fifty adult (2 months old) male Wistar rats weighing between 250-350g, were kept under controlled condition (12hr/12hr light/dark cycles at 25±2°C) in standard PVC cages (five rats in each cage) with free access to water and food (*ad libitum*). All procedures were done in accordance with the Guide for the Care and Use of Laboratory Animals of the National Institutes of Health (NIH; Publication No. 85-23, revised 1985). This experimental research study was confirmed by the regional ethics committee of Tabriz University of Medical Sciences (Ethics approval No. IR.TBZMED.REC.1395.643).

**Study design**

After one-week acclimatization, the animals were randomly divided into 5 groups (n=10 in each); 

1. Control (C) group which did not receive any injection nor procedure.

2. Troxerutin (T) group which received troxerutin 150 mg/kg/day (Merck, Germany) dissolved in distilled water through oral gavage for 4 weeks (Badalzadeh et al., 2015 ▶).

3. Diabetic (DM) group: Diabetes was induced by a single injection of 55 mg/kg streptozotocin (Sigma Chemical Company, St. Louis, MO, USA) intraperitoneally (Alipour et al., 2013 ▶).

4. Troxerutin-receiving DM (DT) group: diabetic rats which received troxerutin 150 mg/kg/day through oral gavage for 4 weeks.

5. Insulin-receiving DM (DI): diabetic rats which were treated with 4 to 6 units of NPH insulin (DarouPakhsh Pharmaceutical MFG co., Iran), once daily for 4 weeks (Choi et al., 2015 ▶). 

Administrations of troxerutin and insulin in DT and DI groups were started after confirmation of DM on the third day after streptozotocin injection. A blood glucose level >250mg/dl was considered a confirmatory evidence of DM. 

**Sampling**

After the intervention period, animals were deeply anesthetized using an intraperitoneal injection of a combination of ketamine and xylazine (80 and 12 mg/kg, respectively). Then, 5 ml blood samples were taken from the inferior vena cava, and centrifuged at 3000 rpm for 10 min at room temperature. Then, serum aliquots were isolated and kept at -80 °C for analysis of insulin levels. Finally, animals were sacrificed by decapitation and both testes were removed. 

**Testicular lipid peroxidation levels and antioxidant activity**

Rats’ right testis were homogenized using cold ice 1.15% KCl to create 10% homogenate. After preparation of supernatant from the testes, oxidative stress parameters were measured. 

**Measurement of malondialdehyde (MDA) levels**

The MDA levels were measured spectrophotometrically based on the thiobarbituric acid reactive substances (TBARS) method (Pourmemar et al., 2017 ▶). Briefly, 0.1 ml of testes homogenate was thoroughly mixed with 0.2 ml of Trichloroacetic acid-thiobarbituric acid-HCl reagent. After centrifugation at 3500 rpm at room temperature for 10 min, the absorbance of the prepared supernatant was determined at 535 nm. MDA concentration was expressed as pmol/mg tissue. 

**Determination of Glutathione peroxidase (GPX) activity**

GPX activity was determined using the method described by Pourmemar (2017) ▶. Accordingly, 0.2 ml of tissue homogenate was mixed with the solutions of 0.2 ml phosphate buffer (0.4 M, pH 7), 0.2 ml glutathione, and 0.1 ml H_2_O_2_ (0.2 M). After 10 min incubation at 37C, 0.4 ml TCA was added to this mixture. Then, GPX concentration was calculated after centrifugation at 3200 rpm for 20 min and expressed as mU/mg protein.

**Determination of Superoxide dismutase (SOD) activity**

SOD activity was determined using RANSOD (Randox Laboratories Ltd, Crumlin, United Kingdom) laboratory kit. The basis for SOD activity measurement is the production of xanthine and xanthine oxidase which results in the production of superoxide radicals. These radicals react with 2-(4-iodophenyl)-3 (4-nitrophenol)-5-phenyl tetrazolium chloride (ITN) and produce red formazan. The activity of SOD was measured by the severity of inhibition of this reaction at 560 nm by a spectrophotometer and expressed as mU/mg protein (Naderi et al., 2015 ▶).

**Apoptosis assay**

After fixation of left testis, 5-μm thick paraffin sections were prepared, deparaffinized, rehydrated and washed with nuclease-free phosphate buffer. Then, *in situ* cell death detection kit, POD (Cat No. 11 6684 817 910; Roche, Mannheim, Germany) was used to detect apoptosis. Through the process of TUNEL (Terminal deoxynucleotidyl transferase dUTP nick end labeling) procedure pretreatments, sections were incubated in methanol containing 0.3% H_2_O_2_ for 30 min at 25ºC to stop endogenous peroxidase activity and then treated with 20 μg/ml proteinase K (Roche, Mannheim, Germany) in PBS for 15min at 37°C before enzymatic labelling. Sections were incubated with 50 µl of TUNEL reaction mixture for 60 min at 37ºC in a dark and humidified chamber, hybridized in POD solution for 30 min, and stained by 3-3'-diaminobenzidine (DAB) for 15 min. Finally, the prepared sections were counter-stained by hematoxylin and apoptotic cells were detected as cells with a dark brown nuclear stain under light microscopy (Wang et al.,2016). To determine the apoptotic index (AI), the number of stained germ cells was counted in 20 tubules for each animal and finally, apoptotic index‐1 (AI‐1) was defined as the number of tubules containing at least one TUNEL-positive cells per 100 tubules and apoptotic index‐2 (AI‐2) was defined as the number of TUNEL‐positive cells per 100 tubules (Shokri et al., 2010 ▶).

**Stereological studies**

After excision and weighing of the left testicle, a small scratch was made on its capsule, and it was placed in 10% formalin to fix its tissue. The tissues were then dehydrated using a graded series of ethanol, cleared in xylene, impregnated in paraffin and embedded in paraffin block. Each block was cut into nine 20-µm thick serial sections and then, cut into 5-µm thickness using a rotary microtome. This process was continued for the whole tissue in each block. The sections on slides were stained with hematoxylin-eosin (H&E). Systematic random sampling protocol was performed for sampling processes and the first section was randomly chosen and 20 to 25-µm thick sections were selected from each block for stereological analysis. Stereological studies were carried out strictly under blind condition and total volume of testis was estimated by point counting method and Cavalieri's principle. The volume fraction of structures, numerical density of cells and also height of germinal epithelium, and length and diameter of seminiferous tubule in testis were estimated using version 9 stereo-investigator system (MBF Bioscience, Micro Bright Field, Inc., Germany) as described previously (Olfati et al., 2018 ▶).

**Statistical analyses**

IBM SPSS^TM^ software (version 22 for Windows; SPSS Inc., USA) was used for all statistical analyses. The data were expressed as mean±standard error of mean (SEM). One-way ANOVA, followed by the *post hoc* Tukey test, was used to compare the study groups. The difference between the groups was considered significant if p<0.05.

## Results

**Serum glucose and insulin levels**

**Figure 1:**
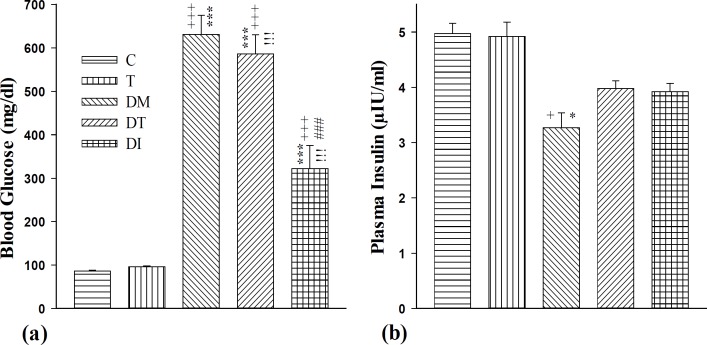
(a) Blood glucose level (mg/dl) and (b) blood insulin level (µIU/ml) levels in control group (C), healthy animals received troxerutin (T), diabetic animals (DM), diabetic animals that received troxerutin (DT) and diabetic animals that received insulin (DI) (n=6 in each group).^ +^ p<0.05 and ^+++^ p<0.001 show statistical differences between control and different groups. ^*^ p<0.05 and ^***^ p<0.001 show statistical differences between troxerutin and different groups. ^iii^ p<0.001 show statistical differences between between DT and DI with DM group. ^###^ p<0.001 shows statistical differences between DI and DT group

Our results revealed that DM, DT and DI groups had significantly higher blood glucose levels compared to the control group (p<0.001 for all cases). Treatment with both troxerutin and insulin significantly lowered serum glucose compared to the DM group (p<0.001 for both cases). However, insulin effect in reducing the blood glucose level was superior to that of troxerutin (p<0.05) (Figure 1a). Moreover, results showed that DM group had significantly lower plasma insulin level compared to both C and T groups (p<0.05 for both cases). Plasma insulin level in DT and DI groups was non-significantly increased compared to the DM group (Figure 1b). 

**Testicular oxidative stress parameters**

**Figure 2:**
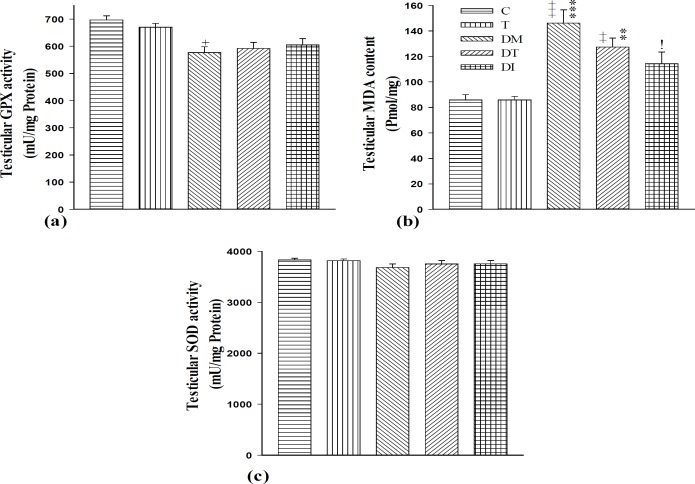
Testicular (a) GPX activity (mU/mg protein), (b) MDA content (pmol/mg), and (c) SOD activity (mU/mg protein) in the testes of control group (C), healthy animals received troxerutin (T), diabetic animals (DM), diabetic animals received troxerutin (DT) and diabetic animals received insulin (DI) (f n=6 in each group). ^+^ p<0.05, ^++^ p<0.01 and ^+++^ p<0.001 show statistical differences between control and different groups. ^**^ p<0.01 and ^***^ p<0.001 show statistical differences between troxerutin and different groups. ^i^ p<0.05 shows statistical differences between DT and DI with DM group. GPX, Glutathione peroxidase; MDA, Malondialdehyde; SOD, Superoxide dismutase

Our results confirmed that DM and DT groups had significantly higher levels of testicular lipid peroxidation, as manifested by higher levels of testicular MDA content compared to group C (p<0.001 for DM and p<0.01 for DT). Also, GPX activity in the testis of diabetic rats was lower than that of the control group (p<0.05). However, testicular SOD activity decreased non-significantly in DM group compared to controls (Figures 2a, b, and c).

Furthermore, administration of insulin to DI group significantly decreased testicular MDA content compared to DM group (p<0.05) although testicular MDA level was non-significantly decreased in DT group compared to diabetic rats. Also, troxerutin and insulin caused non-significant increases in testicular GPX and SOD activities in DT and DI groups compared with the diabetic adult rats (Figures 2a, b, and c). There was no difference between DT and DI groups in testicular MDA content, and GPX and SOD activities. 

**Apoptosis**

**Figure 3:**
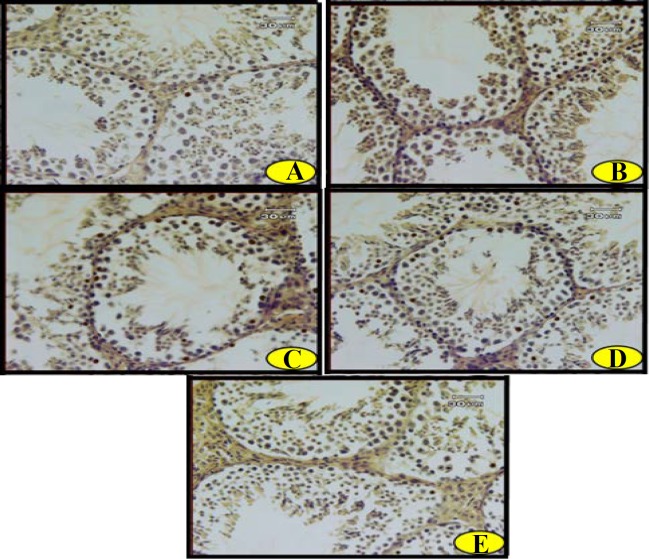
Photomicrographs of apoptotic cells in the testis of (A) control, (B) healthy animals that received troxerutin, (C) diabetic animals, (D) diabetic animals that received troxerutin and (E) diabetic animals that received insulin. Brown-yellow dots display the positive (apoptotic) cells. (Terminal deoxynucleotidyl transferase dUTP nick end labeling (TUNEL); X400)

**Figure 4:**
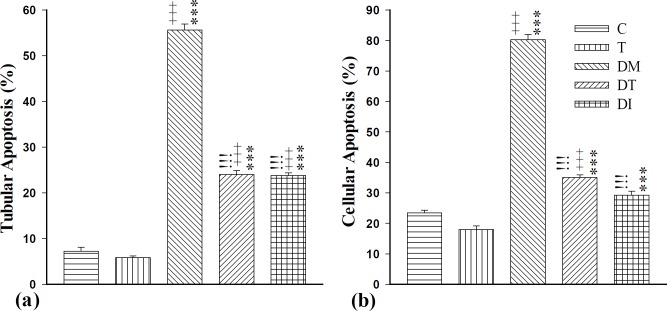
(a) Tubular apoptosis (AI-1) (%) and (b) cellular apoptosis (AI-2) (%) levels in control group (C), healthy animals that received troxerutin (T), diabetic animals (DM), diabetic animals that received troxerutin (DT) and diabetic animals that received insulin (DI) (n=6 in each group). ^+++^ p<0.001 shows statistical differences between control and different groups. ^***^ p<0.001 shows statistical differences between troxerutin and different groups. ^iii^ p<0.001 shows statistical differences between DT and DI with DM group

Analysis of the data revealed a significantly higher tubular apoptosis (AI-1) in DM, DT and DI groups compared to the normal adult rats (p<0.001 for all cases). Moreover, there was significantly increased cellular apoptosis (AI-2) in DM and DT groups compared to group C (p<0.001 for both cases). Administration of troxerutin or insulin decreased both cellular and tubular apoptosis compared to diabetic adult rats (p<0.001). The effects of troxerutin were not found to be superior to those of insulin against tubular and cellular apoptosis (Figures 3A-E and 4a and b).

**Stereological** **findings**

The comprehensive stereological findings of the present study are presented in Table 1. The results showed there were significant changes in seminiferous tubule diameter, germinal epithelium height and volume of seminiferous tubule, germinal epithelium, lumen, capsule, and interstitial in DM group compared to controls (p<0.05 for all cases). Moreover, the number of spermatogonia, spermatocyte, round and elongated spermatid, and Sertoli and Leydig cells decreased in DM group compared to the control group (p<0.05 for all cases). Administration of troxerutin and insulin improved most of these changes compared to diabetic rats (p<0.05 for all cases). No difference was found between troxerutin and insulin groups in this regard. 

## Discussion

Our results showed that chronic administration of troxerutin to type-1 diabetic adult male rats significantly reduced blood glucose level and improved sperm parameters, testicular morphometric and stereological findings, and apoptosis in rat testes. Nevertheless, no significant effect on testicular oxidative stress markers was caused by troxerutin. We also showed that troxerutin effects on the parameters mentioned above, are comparable to those of insulin.

Evidence strongly supports that DM is accompanied by increased oxidative stress and apoptotic cell death in various organs such as the testis (Zhao et al., 2011 ▶; Aybek et al., 2008 ▶). This can be manifested by decreased sperm count, motility and viability, decreased the number of Sertoli and Leydig cells and infertility (Oghbaei et al., 2018 ▶; Keyhanmanesh et al., 2018 ▶; Zavvari Oskuye et al., 2019 ▶). Similarly, the current investigation showed that diabetic rats had higher levels of cellular apoptosis, and testicular lipid peroxidation levels in comparison to the control group. Also, the activity of GPX in these animals was lower than healthy rats. So, DM severely affected the sperm parameters and Sertoli, and Leydig cell counts possibly through the above-mentioned mechanisms.

**Table 1:** Testicular morphometric and stereological findings in control group (C), healthy animals that received troxerutin (T), diabetic animals (DM), diabetic animals that received troxerutin (DT) and diabetic animals that received insulin (DI) (n=6 in each group)

**Study groups**					**Variable**
**DI**	**DT**	**DM**	**T**	**C**	
1.07±0.10	1.27±0.10	1.14±0.20	1.05±0.10	0.91±.05	Testes relative weight (%)
1.15±0.10	0.97±0.10	0.95±0.10	1.10±0.10	0.95±0.5	Left testicle weight (g)
1.77±0.05	1.65±0.10	1.55±0.04	1.71±0.10	1.71±0.04	Left testicle length (cm)
0.94±0.02*	0.82±0.04	0.79±0.01	0.84±0.01	0.89±0.04	Left testicle width (cm)
1.05±0.01	0.96±0.02	0.95±0.13	1.07±0.11	0.99±0.02	Left testicle height (cm)
1200.00±69.20	1088.00±51.61	982.00±34.40*	1212.00±36.00	1110.00±25.10	Left testicle volume (mm^3^)
1006.30±61.00!!!	903.36±45.20!!	682.78±19.00+++***	1089.67±33.15	983.98±28.50	Seminiferous tubule volume (mm^3^)
788.62±62.30!!!	693.44±42.30!!!	356.44±7.92+++***	894.92±31.50	778.60±37.6	Germinal epithelium volume (mm^3^)
217.68±6.35!!!	209.92±4.25!!!	326.34±14.90+++***	194.75±5.60	205.38±10.80	Lumen volume (mm^3^)
29.38±4.54!!	25.96±2.50!!	45.16±2.80+++***	18.25±2.05	20.08±1.72	Capsule volume (mm^3^)
164.32±6.12++**!!!	158.68±6.84++**!!!	254.06±14.42+++***	104.08±3.30	105.94±5.80	Interstitial tissue volume (mm^3^)
17.46±0.80!	17.38±0.85!	14.53±0.60*	17.64±0.30	16.86±0.50	Seminiferous tubule length (m)
423.00±1.22++**!!!	422.00±1.22++**!!!	407.00±2.00+++***	432.00±1.22	435.00±3.53	Seminiferous tubule diameter (µm)
120.00±2.23+++***!!	119.00±1.90+++***!	111.00±1.00+++***	139.00±1.00	138.00±1.22	Germ cell layer height (µm)
36.00±2.07+++***	34.70±1.50+++***	29.46±1.03+++***	59.39±2.82	59.86±0.83	No. of spermatogonia (×10^6^)
170.94±7.41+++***!!!	155.34±6.30+++***!	127.74±6.10+++***	217.68±4.25	217.40±3.75	No. of spermatocyte (×10^6^)
279.44±12.60+++***	275.20±8.40+++***	253.68±11.72+++***	376.98±6.84	377.34±12.65	No. of round spermatid (×10^6^)
306.88±16.60+++***!	274.92±6.50+++***	251.80±14.24+++***	426.26±10.20	430.66±12.60	No. of elongated spermatid (×10^6^)
24.44±1.34++**!!!	24.62±0.40++**!!!	15.49±0.42+++***	31.27±1.20	32.00±1.71	No. of Sertoli cells (×10^6^)
17.46±0.80++**!!!	17.50±0.60++**!!!	11.99±0.64+++***	20.61±0.80	21.09±0.50	No. of Leydig cells (×10^6^)

No; number.

++ ; p<0.01 and

+++ ; p<0.001 show statistical differences between control and different groups.

* ; p<0.05,

** ; p<0.01,

*** and p<0.001 show statistical differences between troxerutin and different groups.

! p<0.05,

!! ; p<0.01,

iii ;p<0.001 show statistical differences between DT and DI with DM group.

Troxerutin, which is commonly known as Vitamin P_4_, is a flavonoid ingredient of coffee, cereal grains, tea, and several fruits (Fan et al., 2009 ▶). Evidence proved the protective effects of troxerutin against various tissue injuries in the brain, liver, and kidney (Badalzadeh et al., 2015 ▶). Moreover, glucose-lowering, anti-inflammatory, anti-apoptotic and anti-oxidant effects of troxerutin were shown in other models (Yu et al., 2017 ▶; Sampath et al., 2014 ▶). In this study, we showed that troxerutin administration meaningfully reduced serum glucose level and non-significantly increased insulin level in diabetic rats; however, these effects were not shown to be superior to those of insulin. Many studies recently found that troxerutin administration improved insulin resistance and decreased serum glucose level in diabetic mice (Lu et al., 2011 ▶; Geetha et al., 2014 ▶). In addition, Sampath et al. in 2014 ▶ showed that troxerutin increased the level of glucose transporter subtype 4 (GLUT-4) and cellular glucose uptake and thus, improved the regulation of blood glucose level in diabetic rats. This study also found that troxerutin, by activation of PI_3_K-Akt pathway, enhanced glucose uptake by the skeletal muscle cells (Sampath et al., 2014 ▶). Another study done by Zhang et al. in 2016 ▶, demonstrated that troxerutin could prevent increased hepatic gluconeogenesis by decreasing hepatic inflammation in high-fat-diet-treated mice (Zhang et al., 2017 ▶).

The present investigation also presented that treatment with troxerutin significantly improved stereological findings such as the number of spermatogonia, spermatocyte, round and elongated spermatid, and Sertoli and Leydig cells in diabetic rats. These effects were found to be comparable to those of insulin. Paucity exists over the effects of troxerutin on sperm parameters in the literature. However, a study done by Elangovan et al. in 2016 ▶, revealed that chronic troxerutin administration to Wistar rats reverses nickel-induced testicular toxicity as manifested by an increase in the testis-organ weight and decreases in oxidative stress (Elangovan et al., 2016 ▶).

The improving effects of troxerutin on the mentioned sperm and stereological findings observed in this study, could be explained by anti-apoptotic properties of this material. The results of this study showed that troxerutin meaningfully decreased the number of TUNEL-positive tubules and cells in the testis of diabetic rats. In line with these data, a study demonstrated that troxerutin administration markedly diminished the number of TUNEL-positive cells, as an indicator of apoptosis, in d-galactose-induced kidney injury in mice (Liu et al., 2010 ▶). Moreover, a study done by Farajdokht et al. in 2017 ▶, revealed that troxerutin treatment significantly decreased the TUNEL-positive cell counts in the d-galactose-induced apoptosis in mice hippocampus (Farajdokht et al., 2017 ▶). In another study done by Mokhtari et al. (2015) ▶ troxerutin decreased the number of TUNEL-positive cells and apoptosis in the diabetic mice myocardium. The authors proposed that phosphorylation of GSK-3β could be a possible mechanism through which, troxerutin reduced apoptosis rate (Mokhtari et al., 2015 ▶).

The improvements of sperm parameters and stereological findings seen in this study, could be also resulted from the antioxidant effects of troxerutin. Although the present study could not show its significant impact, several lines of evidence suggested that troxerutin remarkably decreases oxidative stress (Perumal et al., 2017 ▶; Fan et al., 2008 ▶; Geetha et al., 2017 ▶). Geetha et al. in 2017 ▶, presented that chronic administration of troxerutin decreased lipid peroxidation products and increased enzyme activity of anti-oxidant defence system (i.e. SOD, GPX, and catalase (CAT) activity) in the myocardium of calorie-rich-diet-fed mice (Geetha et al., 2017 ▶). Another study revealed that troxerutin treatment increased SOD, CAT, GPX, glutathione reductase (GR), and glucose-6-phosphate dehydrogenase (G6PD) activities in the nickel-induced testicular toxicity in Wistar rats (Elangovan et al., 2016 ▶). The differences observed between the results of these studies and our results could be due to the variations in the models used and tissues in which oxidative stress was examined. 

In conclusion, the results of this study indicated that troxerutin effectively improves DM-induced testicular dysfunction and sperm parameters in diabetic adult male Wistar rats and this effect was comparable to that of insulin. These impacts could be mediated through troxerutin anti-apoptotic effects. Although previous evidence confirm the anti-oxidant properties of troxerutin, we failed to show such effects in this investigation. In short, this study signified that troxerutin could be regarded as a potential treatment for DM-induced testicular dysfunction and infertility after conducting further studies.
